# Polymorphisms rs693421 and rs2499601 at locus 1q43 and their haplotypes are not associated with primary open-angle glaucoma: a case–control study

**DOI:** 10.1186/s13104-019-4491-x

**Published:** 2019-07-23

**Authors:** Altaf A. Kondkar, Taif A. Azad, Tahira Sultan, Faisal A. Al-Mobarak, Hatem Kalantan, Saleh A. Al-Obeidan

**Affiliations:** 1 Department of Ophthalmology, College of Medicine, King Saud University, P.O. Box 245, Riyadh, 11411 Saudi Arabia; 2 Glaucoma Research Chair in Ophthalmology, College of Medicine, King Saud University, Riyadh, Saudi Arabia

**Keywords:** Chromosome 1q43, Genetic association, Glaucoma, Haplotype, rs693421, rs2499601, Saudi

## Abstract

**Objective:**

The genetic spectrum of primary open-angle glaucoma (POAG) in middle-eastern Saudi’s is still elusive. To this end, we investigated an association between rs693421, rs2499601 and their haplotypes at chromosome 1q43 locus with POAG and its related clinical phenotypes. Genotyping was performed with TaqMan^®^ assays. Haplotypes and their interaction analysis were carried out by SHEsis and SNPStats online tools.

**Results:**

The minor “T” allele frequency of rs693421 was 0.48 in controls and 0.52 in cases (odds ratio (OR) = 1.15, 95% confidence interval (CI) 0.85–1.54, p = 0.368). Similarly, for rs2499601, the minor “C” allele frequency was 0.49 in controls as compared to 0.53 in cases (OR = 1.19, 95% CI 0.89–1.60, p = 0.236). Besides, genotype distribution for both these polymorphisms was also not significant in additive, dominant and recessive models. rs693421 and rs2499601, showed significant linkage disequilibrium (D’ statistics = 0.69, p < 0.001) but haplotype association was non-significant (p = 0.698). The significance did not vary after adjustment to age and sex. No significant genotype association was observed with intraocular pressure, cup/disc ratio and number of anti-glaucoma medication in POAG group. Furthermore, age, sex and genotypes did not contribute any significant risk of POAG in regression analysis. We report no association between rs693421, rs2499601 and their haplotypes with POAG and related phenotypes.

**Electronic supplementary material:**

The online version of this article (10.1186/s13104-019-4491-x) contains supplementary material, which is available to authorized users.

## Introduction

Epidemiological studies in the past suggest that primary open-angle glaucoma (POAG), at least in part, can be caused by heritable factors [1–3], with five to tenfold increased risk in the first-degree relatives and an estimated heritability of 0.81 [4]. However, given the polygenic nature of POAG, its exact genetic etiology still remains elusive. Genetic studies are a critical tool to identify genes and molecular pathways in complex human diseases. Likewise, using a similar approach, population-based genome-wide and candidate gene studies have reported a number of polymorphic variations or loci that have been associated with POAG and/or its quantitative endophenotyes [5, 6].

In an initial study, using a 2-stage genome-wide approach in a group of Japanese POAG patients, Nakano et al. [7] reported 3 genetic loci, harboring six single nucleotide polymorphisms (SNPs), of having significant association with the disease. Of these, 4 intergenic polymorphisms such as rs540782, rs540784, rs693421 and rs2499601 are located on chromosome 1q43. The genes flanking the locus 1q43 include Zona Pellucida glycoprotein 4 (*ZP4*) and Ryanodine Receptor 2 (*RYR2*). *ZP4* plays a role in fertilization and preimplantation development; and genetic variations in *ZP4* are reported to be associated with ovarian disease [8] and glaucoma [7]. Ryanodine receptors (RyRs) are ubiquitous intracellular calcium (Ca^2+^) release channels localized in the plasma membrane of endoplasmic or sarcoplasmic reticulum of many organs, including retina [9]. Given the important physiological role of RyR channel, defect(s) in RyR function, either due to oxidative stress or genetic mutations can render these channels leaky to Ca^2+^ influx. These may thereby produce disease-causing signals and contribute to severe pathologies as those observed in heart failure, muscular dystrophy, diabetes, or neurodegenerative diseases [10, 11]. Also, considering the potential role of calcium channel blockers in treatment of glaucoma [12], *RYR2* may be a potential candidate in POAG.

Replication of case–control association studies in various ethnicities has genetic epidemiological importance, so as to enable future utilization of these genetic biomarkers in evaluation of the disease risk. Association studies using candidate gene approach are a useful tool to identify the genetic contribution of SNPs/loci, each of which may exhibit a relatively small risk but have a significant impact in a population. The genetic spectrum of POAG, especially in middle-eastern Saudi POAG patients, is still unclear. We have previously reported a lack of association between SNPs rs540782 and rs540784 with POAG in a small group of Saudi patients [13, 14]. The present study investigated an association between rs693421, rs2499601 and their haplotypes with POAG or its clinical indices in a much larger and different set of sample cohort of Saudi origin. Besides, there are no other published reports of association of these SNPs (rs693421 and rs2499601) in this population.

## Main text

### Methods

#### Study design and participants

We conducted a retrospective case–control genetic association study. The study followed the principles of the Declaration of Helsinki and had an institutional review board ethics committee approval (# 08-657). Patients (n = 185) and controls (n = 171) were recruited at King Abdul-Aziz University Hospital in Riyadh, Saudi Arabia. The inclusion–exclusion criteria for patients and controls have been detailed elsewhere [15]. Briefly, POAG patients showed (i) optic disk or retinal nerve fiber layer changes; (ii) visual field abnormalities; (iii) bilateral open anterior chamber angles; and (iv) adult onset. Secondary forms of glaucoma and history of steroid usage or ocular trauma were excluded. Controls were > 20 years of age with normal IOP, normal optic disk, open anterior chamber angles and no history of ocular disease(s) or eye surgeries. Subjects refusing to participate were also excluded.

#### Genotyping of rs693421 and rs2499601 at Chr.1q43

DNA samples were genotyped for rs693421 and rs2499601using the TaqMan^®^ genotyping assays C_80796_20 and C__26180887_10, respectively (Applied Biosystems Inc., Foster City, CA, USA; Cat#: 4351379) as described previously [13].

#### Statistical analysis

SPSS version 22 (IBM Inc. Chicago, IIinois, USA) was used to perform statistical tests as indicated. Linkage disequilibrium analysis was conducted using SHEsis online software (http://analysis.bio-x.cn/myAnalysis.php). SNPStats online software (http://bioinfo.iconcologia.net/snpstats/start.htm) was used to construct haplotypes and analyze the interactions with related factors. p < 0.05 (2-sided) was considered statistically significant.

### Results

#### Demographic and clinical characteristics

Except for family history of glaucoma (*p* = 0.019), there was no significant difference in age, gender, systemic co-morbidities and smoking habit between POAG cases and controls (Additional file 1: Table S1).

#### Genotype and allele frequency

rs693421 and rs2499601 did not deviate significantly from the HWE (p > 0.05). For rs693421, the minor “T” allele frequency was 0.48 in controls and 0.52 in cases, but non-significant (OR = 1.15, 95% CI 0.85–1.54, p = 0.368). Besides, genotype distribution was also not significant in additive (Chi^2^ = 1.74, df = 2, p = 0.417), dominant (p = 0.860) and recessive (p = 0.190) models (Table 1). Similarly, for rs2499601, the minor “C” allele frequency was 0.49 in controls as compared to 0.53 in cases (OR = 1.19, 95% CI 0.89–1.60, p = 0.236) with no significant genotype effect in additive (Chi^2^ = 1.69, df = 2, p = 0.429), dominant (p = 0.512) and recessive (p = 0.195) models. In addition, for both the SNPs, the genotype/allele distribution between POAG cases and controls was also non-significant after adjustment for age and sex. There was no significant interaction with sex for rs693421 (p = 0.83) and rs2499601 (p = 0.83); and the significance did not vary after adjustment for age.

**Table 1:** Association analysis of allele frequency and genotype distribution between POAG cases and controls

SNP locus	Gene type	Controls (%)	POAG (%)	OR (95% CI)	p value^a^
Rs693421	GG	43 (25.10	45 (24.3)	1.00	Reference
	GT	91 (53.2)	89 (48.1)	0.93 (0.56–1.56)	0.791
	TT	37 (21.6)	51 (27.6)	1.32 (0.73–2.39)	0.362
	Additive	–	–	–	0.417
	Dominant	–	–	1.05 (0.65–1.69)	0.860
	Recessive	–	–	1.38 (0.85–2.24)	0.190
	G	177 (52.0)	179 (48.0)	1.00	Reference
	T	165 (48.0)	191 (52.0)	1.15 (0.85–1.54)	0.368
	HWE P	0.390	0.616	–	–
Rs2499601	TT	48 (28.1)	47 (25.4)	1.00	Reference
	TC	80 (46.7)	80 (43.2)	1.02 (0.61–1.70)	0.920
	CC	43 (25.1)	58 (31.4)	1.38 (0.78–2.42)	0.265
	Additive	–	–	–	0.429
	Dominant	–	–	1.18 (0.72–1.92)	0.512
	Recessive	–	–	1.36 (0.85–2.16)	0.195
	T	176 (51.0)	174 (47.0)	1.00	Reference
	C	166 (49.0)	196 (53.0)	1.19 (0.89–1.60)	0.236
	HWE P	0.406	0.072		

OR (95% CI): Odds ratio (95% confidence interval), HWE P: Hardy–Weinberg equilibrium p value

^a^Pearson Chi^2^ test

#### Linkage disequilibrium and haplotype association

rs693421 and rs2499601 showed significant linkage disequilibrium as reflected by D’ value (D’ statistics = 0.69, p < 0.001) (Additional file 1: Figure S1). No significant haplotype association was observed (p = 0.698) (Table 2). The significance did not vary after adjustment to age and sex (p = 0.750).

**Table 2:** Haplotype frequencies and association with POAG

Haplotype^a^	Frequency controls	Frequency POAG	Odds ratio (95% confidence interval)	p value
GC	0.079	0.087	1.10 (0.64–1.87)	0.726
GT	0.438	0.397	0.84 (0.62–1.13)	0.267
TC	0.406	0.443	1.16 (0.86–1.56)	0.317
TT	0.076	0.073	0.95 (0.54–1.66)	0.865

Global haplotype association *X*^2^ = 1.43, df = 3, p = 0.698 (Pearson’s)

^a^Haplotypes were sorted in the order of rs693421 and rs2499601

#### Genotype effect on demographic and clinical phenotypes in POAG

Age and gender distribution was not significant for both rs693241 and rs2499601 genotypes. In addition, phenotypes such as intraocular pressure (IOP), cup/disc ratio and number of antiglaucoma medication showed no significant genotype effect (Additional file 1: Table S2). Besides, logistic regression showed that age, sex, rs693241 and rs2496601 could not significantly explain the likelihood of having POAG (Additional file 1: Table S3).

### Discussion

POAG is the second most common type of glaucoma in Saudi Arabia [16]. Despite its high prevalence, the role of genetic factors contributing to the progression and/or development of POAG are largely unknown and thus warrants genetic investigation(s) to identify these factors that may plausibly be associated or contribute to POAG pathogenesis. To this end, evaluation of an association between rs693421, rs2496601 and their haplotypes in a Saudi POAG cohort in this study did not yield any significant relationship with the disease.

Nakano et al. [7] have previously reported a moderate association of polymorphisms rs693241 (OR = 1.48, 95% CI 1.20–1.83, p = 0.00029) and rs2499601 (OR = 1.45, 95% CI 1.17–1.79, p = 0.000589) at locus 1q43 in 1575 Japanese POAG patients. The findings were successfully replicated in stage-2 GWA study. However, except for rs693421 (OR = 1.4 (1.1–1.7), p = 0.0082) in Korean subjects, subsequent studies in a South Indian [17], Afro-Caribbean [18], Japanese [19], the Hong Kong cohort [20], Korean (for SNP rs2499601) [21] and very recently in the Han Chinese population [22] have failed to replicate this association between rs693241, rs2499601 and POAG. Besides, the functional significance of these SNPs or locus to POAG development remains unknown. In contrast to the findings of Nakano et al. [7] and consistent with the findings reported in other population [17, 19–21], no significant genotype/allelic effect of these SNPs in POAG was observed in our study. These conflicting results can be ascribed to factors such as differences in sample size, lack of adequate statistical power or differences in the phenotypes of the sample population included in the study. A comparison of minor allele frequencies of these SNPs in different ethnic groups is shown in Table 3. The minor allele frequencies observed for these variants in the Saudi population (controls) was comparable to the Oriental population’s [7, 19–21], but higher than that reported among the Indian [17] and Afro-Caribbean ethnic groups [18].

**Table 3:** Comparison of rs693421 and rs2499601 minor allele frequency distribution among different ethnic group

Population	SNP	Minor allele frequency^a^		p value	References
		Controls	POAG		
Japan	rs693421	0.450	0.550	0.00029	[7]
	rs2499601	0.460	0.550	0.00058	
India	rs693421	0.357	0.405	0.616	[17]
	rs2499601	0.413	0.482	0.286	
Afro-Caribbean	rs693421	0.291	0.313	0.733	[18]
	rs2499601	0.295	0.271	0.528	
Korea	rs693421	0.476	0.548	0.030	[21]
	rs2499601	0.480	0.533	0.120	
Japan	rs693421	0.498	0.528	0.246	[19]
	rs2499601	0.497	0.530	0.208	
China	rs693421	0.465	0.459	0.98	[20]
	rs2499601	ns	ns	–	
Saudi	rs693421	0.482	0.516	0.368	This study
	rs2499601	0.485	0.530	0.236	

ns: Not studied

^a^ rs693421[T] and rs2499601[C]

The genotypes of adjacent polymorphisms are often highly correlated, referred to as being in linkage disequilibrium, and could be inherited as haplotypes. Haplotypes display an interaction of these polymorphisms and can serve as a maker of disease susceptibility [23]. In complex multifactorial diseases, such as POAG, multiple polymorphisms/loci in the form of haplotypes can be more informative as compared to single allele polymorphism analysis. Hence, to gain additional information we performed association between 1q43 locus haplotypes (for polymorphisms rs693421 and rs2499601) and POAG. The four SNPs (rs540782, rs540784, rs693421 and rs2499601) at chromosome 1q43 have been reported to exhibit strong linkage disequilibrium (D’ = 0.90–1.0) [7, 17], but data on haplotypes with these did not indicate any association with POAG among Indians [17] as opposed to significant association (p < 0.05) among Han Chinese POAG subjects [22]. In our study, significant linkage disequilibrium between rs693421 and rs2499601 was found (D’ = 0.69) but analysis of distribution differences of the four haplotypes in the POAG cases and controls did not show any significant association with the disease. Unfortunately, genotype data for two other SNPs rs540782 and rs540784 at 1q43 were not available for all the subjects included in this study to assess their linkage and/or haplotype association.

Association analysis for any genotype effect of rs693421 and rs2499601 on clinical phenotypes did not provide any significant link. Age and gender showed no significant distribution and clinical phenotypes for glaucoma such as IOP, cup/disc ratio and number of anti-glaucoma medication that serves as markers of disease severity also showed no significant genotype effects in the POAG group.

Similar to Nakano et al. study in 2009, many other recent GWA-studies have reported numerous SNPs in multiple loci/genes in association with POAG and its endophenotypes among European, African and Asian ethnic groups (as reviewed elsewhere [5, 6]). In another recent large-scale study by Shiga et al. [24] in the Japanese population, variants near cyclin-dependent kinase inhibitor 2B-antisense 1 (*CDKN2B*-*AS1*, rs2157719), SIX Homeobox 6 (*SIX6,* rs33912345) and growth arrest-specific 7 (*GAS7*, rs9913911) were found to be associated with POAG. In contrast, with the exception of variant rs10483727 in (*SIX1*/*SIX6*) gene locus [25], our previous studies to identify genetic polymorphisms associated with Saudi POAG patients in genes including caveolin 1/caveolin 2 (*CAV1/CAV2*) [26], atonal homolog 7 (*ATOH7*) [27], (*CDKN2B*) [28], transmembrane and coiled-coil domain 1(*TMCO1*) [29], (*GAS7*) [30] and *ZP4* [13, 14], have yielded negative results indicating that the plausible causal genetic defect(s) in POAG cases of Saudi origin may be different than those from European, African and Asian descents, including the Japanese population. There can be several potential reasons for differences in association findings. The differences could reflect an ethnic-specific genetic etiology of POAG. Furthermore, the differences could be attributed to variations in environmental exposures, clinical variability observed in patients with POAG, variations in the endophenotypic traits related to POAG or other epistatic interactions. Nonetheless, based on our findings and that of reported in other ethnic groups, it appears that polymorphisms rs693421 and rs2499601 at chromosome 1q43 (near *ZP4*/*RYR2* genes), may not have a significant role in POAG pathogenesis and hence, cannot serve as genetic markers to assess the disease risk.

## Limitations

Our study could not replicate the previously observed association of rs693421 and rs2499601 in POAG. The results suggest that these SNPs and their haplotypes at locus 1q43, independently or in relation to other clinical phenotypes, are not associated with POAG and hence may not pose a significant risk of POAG in individuals of Saudi origin. However, the lack of association needs cautious interpretation since the study is limited by its sample size, performed in specific ethnicity and their interaction with other genetic or non-genetic factors cannot be ruled out. Nonetheless, based on the minor allele frequency from 1000 Genomes database or that observed in our current study, the sample size used, showed an estimated power of 90% with a detectable odds per allele of 1.60 (2-sided test) and 1.53 (one-sided test). However, assuming an OR of 1.45 as observed in the Japanese POAG patients [7], our study had only 70% and 71% powers (2-sided test) to detect any significant associations between POAG and rs693421 and rs2499601 polymorphisms, respectively. As in most genetic association studies, in order to detect an odds of ≤ 1.5-fold a much larger sample size would be needed.

## Additional file

**Additional file 1: Table S1.** Demographic and clinical characteristics of POAG cases and controls genotyped for polymorphism rs693421 and rs2499601 included in this study. **Figure S1.** Linkage disequilibrium plot between the two loci. **Table S2.** Genotype effect of rs693421 and rs2499601 on glaucoma specific clinical indices in PAOG cases. **Table S3.** Logistic regression analysis to assess the effect of age, sex, rs693421 and rs2499601 genotypes on POAG outcome.

## Data Availability

The data supporting the conclusions of this article are all presented within the article.

## Abbreviations

*ATOH7*: atonal homolog 7
*CAV1/CAV2*: caveolin 1/caveolin 2
*CDKN2B*: cyclin-dependent kinase inhibitor 2B
CI: confidence interval
GWAS: genome-wide association study
*GAS7*: growth arrest-specific 7
HWE: Hardy–Weinberg Equilibrium
IOP: intraocular pressure
OR: odds ratio
POAG: primary open angle glaucoma
*RYR2*: ryanodine receptor 2
SNP: single nucleotide polymorphism
*SIX1*/*SIX6*: SIX Homeobox 1/SIX Homeobox 6
*TMCO1*: transmembrane and coiled-coil domain 1
*ZP4*: zona pellucida glycoprotein 4
