# Supplementary material for: Polymorphisms rs693421 and rs2499601 at locus 1q43 and their haplotypes are not associated with primary open-angle glaucoma: a case–control study
Source: BMC Res Notes. 2019 Jul 23;12:453. doi: 10.1186/s13104-019-4491-x (PMC6651941; doi:10.1186/s13104-019-4491-x)
Supplement: Supplementary file 1 — Additional file 1: Table S1. Demographic and clinical characteristics of POAG cases and controls genotyped for polymorphism rs693421 and rs2499601 included in this study. Figure S1. Linkage disequilibrium plot between the two loci. Table S2. Genotype effect of rs693421 and rs2499601 on glaucoma specific clinical indices in PAOG cases. Table S3. Logistic regression analysis to assess the effect of age, sex, rs693421 and rs2499601 genotypes on POAG outcome. [file 13104_2019_4491_MOESM1_ESM.doc]

**Additional file**

**Figure S1:** Linkage disequilibrium plot between the two loci

**
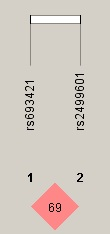
**

**Table S1:** Demographic and clinical characteristics of POAG cases and controls genotyped for polymorphism rs693421 and rs2499601 included in this study

| **Variables** | **Controls**  **(n = 171)**  **No. (%)** | **POAG**  **(n = 185)**  **No. (%)** | ***p* value^a^** |
| --- | --- | --- | --- |
| **Demographic Characteristics** |  |  |  |
| Age in years, mean (±SD) | 59.3 (11.3) | 61.1 (10.5) | 0.114* |
| Male | 95 (55.5) | 103 (55.6) | 0.982 |
| Female | 76 (44.5) | 82 (44.4) | - |
| **Systemic Diseases** |  |  |  |
| Diabetes mellitus | 67 (39.1) | 74 (40.0) | 0.887 |
| Coronary artery disease | 4 (2.3) | 6 (3.2) | 0.606 |
| Hypertension | 57 (33.3) | 70 (37.8) | 0.374 |
| Hypercholesterolemia | 8 (4.6) | 13 (7.0) | 0.348 |
| **Health Awareness / Behavior** |  |  |  |
| Family history of glaucoma | 6 (3.5) | 18 (9.7) | 0.019 |
| Smoking | 14 (8.1) | 20 (10.8) | 0.399 |

^a^Pearson Chi^2^ test, **t*-test

**Table S2** Genotype effect of rs693421 and rs2499601 on glaucoma specific clinical indices in PAOG cases

| **SNP locus** | **Genotype** | **IOP in mmHg, mean (SD)** | **Cup/disc ratio** | **Number of antiglaucoma medications, mean (SD)** |
| --- | --- | --- | --- | --- |
| **Rs693421** | GG | 23.7 (9.0) | 0.76 (0.2) | 1.7 (1.0) |
|  | GT | 23.3 (9.0) | 0.75 (0.2) | 2.0 (1.1) |
|  | TT | 22.7 (9.2) | 0.78 (0.2) | 1.2(0.2) |
|  | p value^a^ | 0.889 | 0.401 | 0.424 |
| **Rs2499601** | TT | 24.5 (9.1) | 0.77 (0.2) | 1.8 (1.1) |
|  | TC | 23.8 (9.6) | 0.74 (0.2) | 1.9 (1.0) |
|  | CC | 21.5 (7.9) | 0.78 (0.2) | 1.8 (1.1) |
|  | p value^a^ | 0.146 | 0.276 | 0.819 |

IOP, intraocular pressure; ^a^Kruskal-Wallis test

**Table S3** Logistic regression analysis to assess the effect of age, sex, rs693421 and rs2499601 genotypes on POAG outcome

| **Variables** | **B** | **S.E.** | **Odds ratio (95% confidence interval)** | **p value** |
| --- | --- | --- | --- | --- |
| Age | 0.016 | 0.010 | 1.02 (0.99 – 1.03) | 0.118 |
| Sex^a^ | -0.001 | 0.218 | 0.99 (0.65 – 1.53) | 0.997 |
| Rs693421^b^ | - | - | - | 0.721 |
| G/T | -0.199 | 0.320 | 0.82 (0.44 – 1.53) | 0.533 |
| T/T | -0.005 | 0.429 | 0.99 (0.43 – 2.30) | 0.991 |
| Rs2499601^c^ | - | - | - | 0.716 |
| T/C | 0.099 | 0.313 | 1.10 (0.59 – 2.04) | 0.752 |
| C/C | 0.317 | 0.404 | 1.37 (0.62 – 3.03) | 0.433 |
| Constant | -0.898 | 0.660 | 0.41 | 0.174 |

^a^Female as reference, ^b^G/G as reference, ^c^T/T as reference
